# The Impact of Surgical Repair on Restlessness in Infants with Non-Incarcerated Inguinal Hernias: A Prospective Study

**DOI:** 10.3390/jcm14041105

**Published:** 2025-02-09

**Authors:** Ortal Schaffer, Ori Blich, Alon Yulevich, Eleonora Niazov, Yaron Armon, Osnat Zmora

**Affiliations:** 1 Faculty of Medical and Health Sciences, Tel Aviv University, Tel Aviv 6997801, Israel; ortal.schaffer@gmail.com (O.S.); gadi177@walla.co.il (E.N.); 2 Department of Pediatric Surgery, Shamir Medical Center, Zerifin 7073001, Israel; 3 Pediatric Surgery Department, Shaare Zedek Medical Center, Jerusalem 9103102, Israel; orib@szmc.org.il (O.B.); yaronarmon@szmc.org.il (Y.A.); 4 Department of Pediatric Surgery, Ziv Medical Center, Safed 1311001, Israel; alony@ziv.gov.il

**Keywords:** inguinal hernia, restlessness, infants

## Abstract

**Background/Objectives**: Pediatric inguinal hernias are usually described as asymptomatic unless they become incarcerated. Our aim was to evaluate possible restlessness associated with non-incarcerated inguinal hernias in infants. **Methods**: We performed a prospective multi-center cohort study that included infants, up to 18 months of age, with non-incarcerated inguinal hernias. Restlessness was evaluated by caregivers before (“Pre”) and after (“Post”) hernia repair using two scales, the soothability section of the Infant Behavioral Questionnaire (IBQ) and the Parents’ Restlessness Score (PRS) on a 1–5 scale (5—highest degree of restlessness), and then compared to matched healthy controls. The change in restlessness after surgery was evaluated by Parents’ Change in restlessness Score (PCS) and the difference between Pre- and Post-scores (Δ). A subgroup analysis for patients with Pre-PRS ≥ 3 was performed. Demographic and clinical characteristics were collected, and possible associations with levels of restlessness were evaluated. **Results**: Seventy-nine infants, median corrected age 2.5 (1.6–4.8) months, were included in this study during June 2022 to July 2024. Infants with inguinal hernias were found to suffer significant restlessness as compared to controls [Pre-PRS = 3 (2–4) vs. 2 (2–2), respectively, *p* < 0.001]. After hernia repair, PRS restlessness scores improved [ΔPRS = 1 (0–2)] to the level of controls (Post-PRS = 2 (1–3), *p* = 0.5). The difference in IBQ scores between hernia patients prior to repair and control patients was not statistically significant [3.2 (2.3–4.1) vs. 3.1 (2.3–4.1), respectively, *p* = 0.28], although both Post-PRS and Post-IBQ scores significantly improved as compared to Pre-PRS and Pre-IBQ scores [2 (1–3) and 2.8 (1.7–3.6) vs. 3 (2–4) and 3.2 (2.3–41), *p* < 0.001 and *p* = 0.005, respectively]. Fifty-two patients (66%) had Pre-PRS ≥ 3 and demonstrated a more pronounced improvement in restlessness following surgery [ΔPRS = 1.5 (1–2)]. Patients who had Emergency Department visits prior to hernia repair demonstrated both increased baseline restlessness and more pronounced improvement following repair as compared to patients with no visits (Pre-PRS = 3 (3–4) vs. 3 (2–3), *p* = 0.03; ΔPRS = 1.5 (1–2) vs. 0 (0–1), *p* < 0.01; ΔIBQ = 0.7 (0.02–1.45) vs. 0.12 (−0.5–1), *p* = 0.03). **Conclusions**: Non-incarcerated inguinal hernias in infants are associated with significant restlessness in most cases. Restlessness resolved after surgical repair.

## 1. Introduction

The most common causes of crying, restlessness, and irritability in infants are unmet basic needs, such as the desire for contact from the parent, hunger, or fatigue. In more severe cases, restlessness points to medical problems that require immediate medical attention and possibly urgent intervention, such as ear infections, intussusception, incarcerated hernia, etc. There can also be “intermediate” conditions, such as crying, due to gas in the digestive system and overfeeding [1,2].

While non-incarcerated inguinal hernias in children are usually described in the literature as asymptomatic lumps, incarcerated hernias are characterized in infants by fussiness, restlessness, and irritable crying, which parents find difficult to soothe. Older children, who are already verbal, will complain of severe pain [3,4]. Irreducible but not strangulated ovaries are also described in the literature as asymptomatic groin masses [5].

When an inguinal hernia is diagnosed in a child, surgical repair is recommended. When the hernia is incarcerated, manual reduction is attempted. If unsuccessful, urgent surgery should be performed. Traditionally, an open surgical hernia repair has been the method of choice, with high success rates and a low risk of complications. The development of minimally invasive pediatric surgery in the last few decades has wide acceptance for hernia repair, with numerous new laparoscopic techniques. In comparison with open surgery, the laparoscopic approach is equivalent in terms of surgical time, length of hospital stay, and recurrence rates and allows for the opportunity to explore and repair the contralateral side, preventing metachronous hernia [6].

Our clinical experience has shown that, sometimes, the parents of infants with inguinal hernias that are not incarcerated describe their children as irritable and restless, with subsequent calmer and more peaceful behavior after hernia repair.

Our study aim was to test our observation that infants with non-incarcerated inguinal hernias suffer from restlessness, which resolves with hernia repair, and to challenge the existing paradigm that non-incarcerated inguinal hernias in infants are asymptomatic. Thus, improved parental education could be provided, while the need to search for other causes for restlessness in infants with inguinal hernias might be obviated.

## 2. Materials and Methods

### 2.1. Patients

We conducted a prospective cohort study of all infants younger than 18 months who underwent open repair of inguinal hernias in three medical centers (Shamir Medical Center, Shaare Zedek Medical Center, and Ziv Medical Center) during June 2022 to July 2024. Infants who presented with incarcerated hernias at any time were excluded. A control group of healthy infants younger than 18 months old, matched by corrected age (±1 month), sex, and prematurity vs. term status, was also evaluated.

### 2.2. Ethical Aspects

This study was approved by the institutional review boards of the three participating medical centers in accordance with the Declaration of Helsinki as revised in 2013, approval #ASF-0052-22; SZMC-0148-23; ZIV-0107-22.

### 2.3. Study Outcomes

Parents were given questionnaires that included three scales used to measure restlessness. The first scale was the soothability section of the validated Infant Behavioral Questionnaire (IBQ) [7], which was filled out by the infant’s regular caregivers prior to surgery (Pre-IBQ), after surgery (Post-IBQ), and in the control group (Ctr-IBQ). In the second scale, the caregivers were asked to score how often their child was restless on a 1–5 scale: Parents’ Restlessness Score (PRS): 1—never/almost never; 2—seldom; 3—half the time; 4—often; 5—always/almost always. The score was used prior to surgical repair (Pre-PRS); after surgical repair, either on a post-operative clinic visit or via telephone interview (Post-PRS); and in the control group (Ctr-PRS). In the third scale, the parents of infants with inguinal hernias were asked to score their impression of the change in their child’s restlessness following surgery using the Parents’ Change in restlessness Score (PCS): 1—significantly improved; 2—mildly improved; 3—unchanged; 4—mildly worse; 5—markedly worse.

The primary outcome of this study was the level of restlessness, as measured by PRS, after surgery compared to prior to surgery. The secondary outcomes were the level of restlessness measured by IBQ, post-surgery compared to prior to surgery; PCS; and level of restlessness in infants with inguinal hernias compared to healthy infants. A sample size of 21 hernia patients was determined to provide 80% power to detect a clinically meaningful improvement of at least 1 point in the mean or median PRS after surgery, as compared to before surgery, assuming an alpha level of 0.05 and a standard deviation of 1.15 (based on a uniform distribution of scores between 1 and 5).

### 2.4. Study Design

Demographic and clinical data were gathered from the electronic hospital files for patients with inguinal hernias and included gestational age, chronological and corrected age at the time of surgery, sex, hernia laterality, pre-operative Emergency Department (ED) visits, presence of irreducible ovaries in female patients, and intra-operative/post-operative complications. The follow-up time from surgery to the post-operative questionnaire was calculated.

Possible associations between various demographic and clinical characteristics and pre-operative scores, post-operative scores, PCSs, and the calculated differences (Δ) between pre-operative and post-operative scores were examined. Correlations between IBQ and PRS scores were evaluated as well as correlations between PCS and the calculated differences (Δ) between pre-operative and post-operative IBQ and PRS.

The parents of the healthy infants in the control group were asked to fill out a questionnaire consisting of the soothability section of the IBQ and PRS scale. The IBQ and PRS scores were compared between the study group and the control group.

### 2.5. Statistical Analysis

Categorical variables were reported as frequency and percentage. Continuous variable distributions were evaluated using histograms and, since all variables were not normally distributed, were reported as the median and interquartile range (IQR). Spearman’s rank correlation coefficients [8] were used to study the associations between continuous and ordinal variables. The Mann–Whitney test was used to study the associations between continuous and ordinal variables and dichotomous variables. The Wilcoxon test was used to compare the hernia group before and after surgery and the hernia and control groups. A sub-analysis was performed for the study participants with a pre-surgical PRS score ≥ 3 (significant pre-operative restlessness). All statistical tests were two sided. *p* < 0.05 was considered statistically significant. SPSS software, version 28 (IBM SPSS statistics, IBM Corp, Armonk, NY, USA) was used to perform the statistical analysis.

## 3. Results

During May 2022 to July 2024, 79 patients were prospectively included in this study from three medical centers (center I—64 patients, center II—9 patients, center III—6 patients). Fifty-five patients (69.6%) were born on term, the median birth week was 38 (35–39), the median corrected age was 2.5 (1.6–4.8) months, and 57 (72.2%) patients were male. The hernia was unilateral in 66 (83.5%) patients, 28 (35.4%) patients had at least one ED visit prior to hernia repair, and 13/22 (59.1%) female patients presented with an irreducible ovary. Complications were documented in only three patients: one laryngospasm during recovery from anesthesia, one adenovirus infection discovered on Post-Operative Day (POD) 2, and one readmission with bronchiolitis on POD 2.

### 3.1. Correlations Between the Different Restlessness Scores

A moderate correlation was found between ΔPRS and PCS (R = −0.538, *p* < 0.001) and between ΔPRS and ΔIBQ (R = 0.552, *p* < 0.001). A weak correlation was found between ΔIBQ and PCS (R = −0.381, *p* < 0.001). A weak correlation was found between Pre-PRS and Pre-IBQ (R = 0.325, *p* = 0.003) and between Post-PRS and Post-IBQ (R = 0.393, *p* = 0.000).

### 3.2. Comparison to Control

Seventy-six pairs of hernia patients and control participants were found via exact matching by corrected age (±1 month), sex, and term versus prematurity status. Table 1 presents the comparison between the matched hernia and control groups. Pre-operatively, PRS in infants with inguinal hernias was found to be statistically significantly higher than in matched infants without inguinal hernias, pointing to increased restlessness in infants with inguinal hernias. Following surgical repair, PRS in infants with inguinal hernias was similar to PRS in matched infants without inguinal hernias, pointing to normalization of infant behavior. IBQ scores were similar between the control group and hernia group.

### 3.3. Comparison of Hernia Patients Before and After Hernia Repair

**Figure 1:**
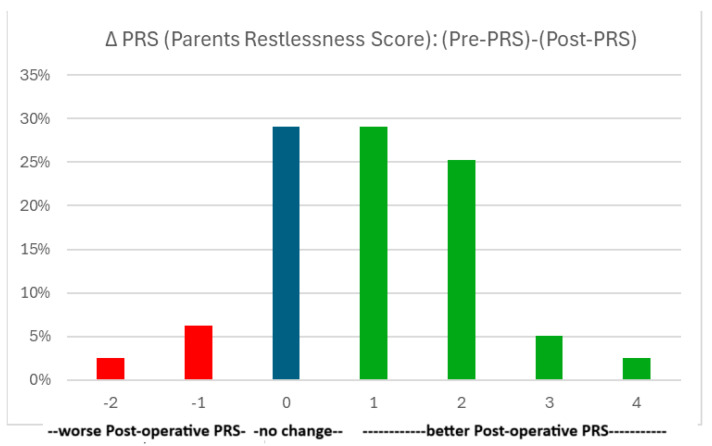
Distribution of ΔPRS score amongst all patients with inguinal hernias.

**Figure 2:**
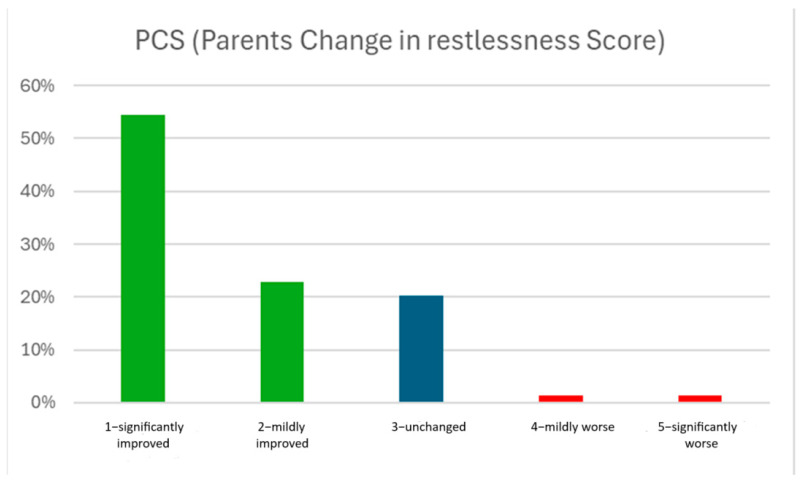
Distribution of PCS scores among all patients with inguinal hernias.

Post-operative questionnaires were filled out after a median of 28 (21–40) days. There was a significant difference between median Pre-PRS and Post-PRS scores [3 (2–4) vs. 2 (1–3), respectively, *p* < 0.001]. In 49 patients, PRS improved after hernia repair; in seven patients, it worsened; and in 23 patients, it remained unchanged. The median difference between Pre-PRS and Post-PRS (ΔPRS) was 1 (0–2) point (Figure 1). The parents’ estimation of the change in their child’s restlessness following surgery (PCS) also demonstrated significant improvement after surgery, with the median PCS = 1 (1–2) (Figure 2). The median pre-IBQ score was 3.2 (2.3–4.1), and the median post-IBQ score was 2.8 (1.7–3.6), *p* = 0.005. The median difference between the Post-IBQ score and Pre-IBQ score was 0.3 (−0.3–1.1).

### 3.4. Association of Patient Factors with Restlessness

Table 2 presents the results of the analysis performed to find possible associations between different patient factors and restlessness scores in the hernia group, before and after surgery, and with changes in restlessness following surgery. Of note, pre-operative ED visits were found to be significantly associated with both higher pre-operative PRS and greater improvement in restlessness following hernia repair, as measured by all three scores (ΔPRS, PCS, and ΔIBQ). Also of note, bilateral hernias, non-reducible ovaries, and time post-op were not associated with any restlessness scores. Higher pre-operative PRS was found to be significantly associated with a higher post-operative PRS, strongly correlated with greater improvement in PRS, and weakly correlated with greater improvement as measured by PCS and by ΔIBQ.

### 3.5. Sub-Analysis of Patients with Pre-PRS ≥ 3 (N = 52)

The majority of this study’s cohort (52/79, 66%) was described as suffering significant restlessness prior to surgical repair, with Pre-PRS ≥ 3. In this subgroup, the median Pre-operative PRS was 3 (3–4), and the median Post-operative PRS was 2 (1–3), *p* < 0.001. Amongst the patients in this subgroup, PRS improved after hernia repair in 42 patients, worsened in one patient, and remained unchanged in nine patients. The median difference between Pre-PRS and Post-PRS (ΔPRS) was 1.5 (1–2) points, while the median ΔPRS for all hernia patients was 1 (0–2). The parents’ estimation of the change in their child’s restlessness following surgery (PCS) was the same as the PCS for all hernia patients [PCS = 1 (1–2), significant improvement]. The median pre-operative IBQ score was 3.6 (3.0–4.1), and the median post-operative IBQ score was 3 (1.6–3.7), *p* < 0.001. The median ΔIBQ was 0.51 (−0.1–1.3), while the median ΔIBQ for all hernia patients was 0.3 (−0.3–1.1).

## 4. Discussion

In this study, we demonstrated, for the first time, that infants with non-incarcerated inguinal hernias suffer from significant restlessness compared to healthy infants, with most infants reported by their parents to be restless at least half the time. We further demonstrated that surgical repair resulted in resolution of restlessness and that the more restless infants improved the most. Inguinal hernia is one of the most common surgical pathologies worldwide, affecting both children and adults [9,10,11]. As reported in the literature, inguinal hernias in adults are almost always symptomatic, and the only definitive treatment is surgery [12,13]. The earliest presentation in adults is usually a groin mass, with or without discomfort. Subsequently, sudden stabbing pain in the groin area can occur, often triggered by physical exertion or exercise. More commonly, a dull, aching pain radiates down to the scrotum [14]. A randomized controlled trial, comparing “watchful waiting” to surgical treatment for minimally symptomatic or asymptomatic adults with inguinal hernias, assessed complaints using a 4-point pain/discomfort score (0 = asymptomatic, 4 = severe pain/discomfort). The trial demonstrated that asymptomatic or mildly symptomatic adults, initially assigned to the watchful waiting group, frequently transitioned to the surgery group over time, with developing pain/discomfort being the primary reason for crossover [13,15,16].

In contradistinction, it is generally thought that pediatric inguinal hernias are usually not associated with pain unless they become incarcerated or strangulated [3,4]. Children typically present with an asymptomatic lump or swelling in the groin, scrotum, or labia. This is usually noticed by a parent or caregiver and tends to change in size with activities such as coughing or crying [9,11,17]. However, in babies, it can be difficult to determine if the lump is more noticeable with crying, or whether in fact, the lump itself causes pain (and therefore the crying) [18].

Our study demonstrates that infants with non-incarcerated inguinal hernias exhibit more restlessness as compared to healthy infants without hernias. Notably, this restlessness resolves with surgical repair. These findings are supported not only by the caregivers’ estimation of behavioral changes post-surgery but also by the calculated difference between pre- and post-surgery restlessness scores, both PRS and IBQ. However, the IBQ scores were not significantly different between infants with or without inguinal hernias.

In this study, we used several measuring scales for restlessness and for the change in restlessness in order to compile reliable results. We also tested correlations between the different scales. Despite the moderate correlations that were found between the different measurements of change in restlessness, between ΔPRS and PCS and between ΔPRS and ΔIBQ, weak correlations were found between ΔIBQ and PCS, between Pre-PRS and Pre-IBQ, and between Post-PRS and Post-IBQ. This is likely because the IBQ primarily evaluates soothability, as a temperament related behavior, as opposed to PRS, which measures frequency of restlessness, reflecting pain or discomfort [19]. Therefore, the evaluation of restlessness, which reflects pain or discomfort in healthy infants and in infants with hernias, is probably better achieved by the Parents Restlessness Score.

One of the interesting findings in our study is the increased restlessness in infants who had visited the ED prior to hernia repair (without findings of hernia incarceration, as these cases were excluded from participation in this study). This reinforces the PRS score as a measure of the child’s restlessness. The parents who brought their child to the ED probably did so because of severe restlessness, as supported by the higher PRSs of these children.

In our study, female infants with irreducible ovaries also suffered from restlessness in a similar way to female patients with reducible hernias and to male patients. Irreducible ovaries in inguinal hernias are described in the literature as presenting with asymptomatic groin masses [5]. These are not considered strangulated or treated as an emergency [20,21,22]. Our findings in female infants with irreducible ovaries further highlight the restlessness as a possible symptom in all infants with inguinal hernias, including female patients with irreducible ovaries. These findings also reinforce the current recommendation in the literature to treat patients with irreducible ovaries on the same timeline as patients with reducible hernias.

Most of the study patients (66%) suffered significant restlessness. These patients demonstrated a more pronounced improvement after repair, with higher ΔPRS and higher ΔIBQ values compared to these differences in the entire study cohort. These findings are important for parental education, emphasizing that not all infants with inguinal hernias suffer from restlessness. However, those who suffer more significant restlessness are expected to improve more.

Although various tools are available for assessing acute pain and discomfort in infants, including neonates [23,24,25], it is very difficult to assess pain and discomfort in infants in chronic home settings. It is possible that recurrent or chronic pain and discomfort in infants manifest and are conceived by parents as restlessness and irritability. Our findings, therefore, suggest that infants with inguinal hernias might suffer pain and discomfort as a result of their hernias in a similar way to adults. Older children were not assessed in our study; however, the literature does not support pain and discomfort in older children with inguinal hernias unless their hernia is incarcerated. It is possible that hernias in infants are distinct from hernias in older children and represent a more severe variant that causes symptoms.

The data presented in this study might have several clinical implications. The first concerns the timing of repair. The timing of repair in infants, especially premature infants, is influenced by increased surgical and anesthetical complications on the one hand [26,27] and by higher incidence of incarceration on the other hand [28]. Our data might add another argument for repairing infants with significant restlessness (PRS ≥ 3) sooner rather than later. In addition, the data presented in this study contribute to parental education regarding the possible association between their child’s hernia and restlessness, with anticipation for probable resolution after surgical repair. Lastly, the data provided can assist physicians to limit concerns for other causes of restlessness and can obviate the need for extensive workup.

This study has several limitations. While it is a prospective study conducted across three institutions, the sample size remains small. In addition, there is an unequal distribution of patients between the participating centers, which can cause population bias and limit the globalization of the results. Another limitation, which is not specific to this study, is the lack of valid tools to assess pain and discomfort in infants in chronic home settings, which probably explains the medium or weak correlations found between the different instruments used in this study to measure the results.

## 5. Conclusions

Non-incarcerated inguinal hernias in infants are associated with significant restlessness in the majority of cases. Restlessness resolves after surgical repair and normalizes to a healthy control level.

## Figures and Tables

**Table 1:** Comparison of matched hernia and control groups.

	Hernia N = 76	Control N = 76	*p*
male:female	55:21	55:21	NA
term:preterm	53:23	53:23	NA
Corrected age in months (median and IQR)	2.6 (1.6–4.2)	2.8 (1.7–4.6)	0.98
PRS (median and IQR)	Pre: 3 (2–4)	2 (2–2)	<0.001
	Post: 2 (1–3)	2 (2–2)	0.5
IBQ (median and IQR)	Pre: 3.2 (2.3–4.1)	3.1 (2.3–4.1)	0.28
	Post: 2.9 (1.7–3.7)	3.1 (2.3–4.1)	0.1

IBQ—Infant Behavioral Questionnaire; IQR—Inter Quartile Range; NA—Not Applicable; Post—Post-surgical; Pre—Pre-surgical; PRS—Parents Restlessness Score.

**Table 2:** Association of patient characteristics with restlessness scores.

Patient Characteristics	Pre-PRS		Post-PRS		ΔPRS		PCS		ΔIBQ	
	Median (IQR)	*p*	Median (IQR)	*p*	Median (IQR)	*p*	Median (IQR)	*p*	Median (IQR)	*p*
male:female	3 (2–4):3 (1.75–3)	0.094	**2 (1–3):1 (1–2)**	**0.04**	1 (0–2):1.5 (0–2)	0.51	1 (1–2.5):1 (1–2)	0.31	0.25 (−0.46–1.23):0.49 (−0.26–0.87)	0.86
preterm:term	3.2 ± 1.2:2.8 ± 1.2	0.07	1.8 ± 0.9:2 ± 0.9	0.36	**1.4 ± 1.4:0.7 ± 1.1**	**0.03**	1.7 ± 0.9:1.7 ± 0.9	0.92	1.7 ± 1.3:1.7 ± 0.9	0.27
unilat:bilat	3 (2–3.25):3 (3–4.5)	0.13	2 (1–3):2 (1–3)	0.83	1 (0–2):2 (0.5–2)	0.06	1 (1–2.25):1 (1–2)	0.49	0.27 (−0.29–1.14):0.36 (−0.94–1.08)	0.71
no ED:yes ED	**3 (2–3):3 (3–4)**	**0.03**	2 (1–3):2 (1–2)	0.25	**0 (0–1):1.5 (1–2)**	**<0.001**	**2 (1–3):1 (1–2)**	**0.03**	**0.12 (−0.5–1):0.7 (0.02–1.45)**	**0.03**
reducible:non-reducible (females)	3 (1.5–3.5):2 (1.5–3)	0.51	1 (1–2):1 (1–2)	0.69	2 (0–2):1 (0–2)	0.56	1 (1–2.5):1 (1–2)	0.95	0.12 (−0.25–0.83):0.62 (−0.27–0.87)	0.69
	Correlation coefficient	*p*	Correlation coefficient	*p*	Correlation coefficient	*p*	Correlation coefficient	*p*	Correlation coefficient	*p*
Corrected age, months	**−0.23**	**0.04**	−0.14	0.23	−0.14	0.23	0.08	0.46	−0.11	0.33
Time post-op	−0.03	0.82	−0.19	0.08	0.09	0.39	0.13	0.25	−0.0	0.99
Pre-PRS	1.00	NA	**0.36**	**<0.001**	**0.67**	**<0.001**	**−0.37**	**<0.001**	**0.27**	**0.02**

Bolded numbers signify statistically significant values; Bilat—bilateral; ED—Emergency Department visit/s; IQR—Inter Quartile Range; NA- not available; PCS—Parents’ Change in restlessness Score; Post-PRS—Post-operative Parents Restlessness Score; Pre-PRS—Pre-operative Parents Restlessness Score; Unilat—unilateral; post-op—post-operative; ΔIBQ—The difference between pre-operative and post-operative Infant Behavioral Questionnaire Soothability score; ΔPRS—The difference between Pre-PRS and Post-PRS.

## Data Availability

The data presented in this study are available on request from the corresponding author.

## Abbreviations

The following abbreviations are used in this manuscript:

ED	Emergency Department
IBQ	Infant Behavioral Questionnaire
IQR	Interquartile Range
PCS	Parents’ Change in restlessness Score
POD	Post-Operative Day
PRS	Parents’ Restlessness Score

## References

[1] Ismail J., Nallasamy K. (2017). Crying Infant. Indian J. Pediatr..

[2] Trocinski D.R., Pearigen P.D. (1998). The crying infant. Emerg. Med. Clin. North. Am..

[3] Lao O.B., Fitzgibbons R.J., Cusick R.A. (2012). Pediatric inguinal hernias, hydroceles, and undescended testicles. Surg. Clin. N. Am..

[4] Ein S.H., Njere I., Ein A. (2006). Six thousand three hundred sixty-one pediatric inguinal hernias: A 35-year review. J. Pediatr. Surg..

[5] Patel B., Zivin S., Panchal N., Wilbur A., Bresler M. (2014). Sonography of female genital hernias presenting as labia majora masses. J. Ultrasound Med..

[6] Pogorelić Z., Anand S., Križanac Z., Singh A. (2021). Comparison of Recurrence and Complication Rates Following Laparoscopic Inguinal Hernia Repair among Preterm versus Full-Term Newborns: A Systematic Review and Meta-Analysis. Children.

[7] Rothbart M.K. (1981). Measurement of temperament in infancy. Child. Dev..

[8] Schober P., Boer C., Schwarte L. (2018). Correlation Coefficients: Appropriate Use and Interpretation. Anesth. Analg..

[9] Yeap E., Pacilli M., Nataraja R.M. (2020). Inguinal hernias in children. Aust. J. Gen. Pract..

[10] Kingsnorth A., LeBlanc K. (2003). Hernias: Inguinal and incisional. Lancet.

[11] Hutson J.M., O’Brien M., Beasley S.W., Teague W.J., King S.K. (2015). Jones’ Clinical Paediatric Surgery.

[12] HerniaSurge Group (2018). International guidelines for groin hernia management. Hernia.

[13] Fitzgibbons R.J., Ramanan B., Arya S., Turner S.A., Li X., Gibbs J.O., Reda D.J., Investigators of the Original Trial (2013). Long-term results of a randomized controlled trial of a nonoperative strategy (watchful waiting) for men with minimally symptomatic inguinal hernias. Ann. Surg..

[14] Berndsen M.R., Gudbjartsson T., Berndsen F.H. (2019). Inguinal hernia—Review. Laeknabladid.

[15] Van den Dop L.M., Van Egmond S., Heijne J., van Rosmalen J., de Goede B., Wijsmuller A.R., Kleinrensink G.J., Tanis P.J., Jeekel J., Lange J.F. (2023). Twelve-year outcomes of watchful waiting versus surgery of mildly symptomatic or asymptomatic inguinal hernia in men aged 50 years and older: A randomised controlled trial. EClinicalMedicine.

[16] Chung L., Norrie J., O’Dwyer P.J. (2011). Long-term follow-up of patients with a painless inguinal hernia from a randomized clinical trial. Br. J. Surg..

[17] Bosquet E.M., White M.T., Hails K., Cabrera I., Wright R.J. (2016). The Infant Behavior Questionnaire-Revised: Factor structure in a culturally and sociodemographically diverse sample in the United States. Infant. Behav. Dev..

[18] Abdulhai S.A., Glenn I.C., Ponsky T.A. (2017). Incarcerated Pediatric Hernias. Surg. Clin. N. Am..

[19] Ohkura T., Kumori K., Kawamura T., Manako J., Ishibashi S., Funabashi N., Tajima Y. (2022). Association of pediatric inguinal hernia contents with patient age and sex. Pediatr. Int..

[20] Dreuning K.M., Barendsen R.W., van Trotsenburg A.P., Twisk J.W., Sleeboom C., van Heurn L.E., Derikx J.P. (2020). Inguinal hernia in girls: A retrospective analysis of over 1000 patients. J. Pediatr. Surg..

[21] Hirabayashi T., Ueno S., Hirakawa H., Tei E., Mori M. (2017). Surgical Treatment of Inguinal Hernia with Prolapsed Ovary in Young Girls: Emergency Surgery or Elective Surgery. Tokai J. Exp. Clin. Med..

[22] Esposito C., Gargiulo F., Farina A., Del Conte F., Cortese G., Servillo G., Escolino M. (2019). Laparoscopic Treatment of Inguinal Ovarian Hernia in Female Infants and Children: Standardizing the Technique. J. Laparoendosc. Adv. Surg. Tech. A.

[23] Smith-Parrish M., Vargas Chaves D.P., Taylor K., Achuff B.J., Lasa J.J., Hopper A., Ramamoorthy C. (2022). Analgesia, Sedation, and Anesthesia for Neonates With Cardiac Disease. Pediatrics.

[24] Castagno E., Fabiano G., Carmellino V., Cerchio R., De B., Lauria B., Mercurio G., Coscia A., Ponte G., Bondone C. (2022). Neonatal pain assessment scales: Review of the literature. Prof. Inferm..

[25] Mencía S., Alonso C., Pallás-Alonso C., López-Herce J. (2022). Maternal And Child Health And Development Network Ii Samid Ii. Evaluation and Treatment of Pain in Fetuses, Neonates and Children. Children.

[26] Cho Y.J., Kwon H., Ha S., Kim S.C., Kim D.Y., Namgoong J.M., Nam S.H., Lee J.Y., Jung E., Cho M.J. (2023). Optimal timing for inguinal hernia repair in premature infants: Surgical issues for inguinal hernia in premature infants. Ann. Surg. Treat. Res..

[27] Sacks M.A., Neal D., Pairawan S., Tagge E.P., Hashmi A., Islam S., Khan F.A. (2023). Optimal Timing of Inguinal Hernia Repair in Premature Infants: A NSQIP-P Study. J. Surg. Res..

[28] Zamakhshary M., To T., Guan J., Langer J.C. (2008). Risk of incarceration of inguinal hernia among infants and young children awaiting elective surgery. Can. Med. Assoc. J..

